# Image Derived Input Function for [^18^F]-FEPPA: Application to Quantify Translocator Protein (18 kDa) in the Human Brain

**DOI:** 10.1371/journal.pone.0115768

**Published:** 2014-12-30

**Authors:** Rostom Mabrouk, Pablo M. Rusjan, Romina Mizrahi, Mark F. Jacobs, Yuko Koshimori, Sylvain Houle, Ji Hyun Ko, Antonio P. Strafella

**Affiliations:** 1 Research Imaging Centre, Centre for Addiction and Mental Health (CAMH), Toronto, Ontario, Canada; 2 Morton and Gloria Shulman Movement Disorder Unit, E.J. Safra Parkinson Disease Program, Toronto Western Hospital, UHN, University of Toronto, Toronto, Canada; 3 Division of Brain, Imaging and Behaviour, Systems Neuroscience, Toronto Western Research Institute, UHN, University of Toronto, Toronto, Ontario, Canada; 4 Department of Human Anatomy and Cell Science, University of Manitoba, Winnipeg, Manitoba, Canada; 5 Department of Psychiatry, University of Toronto, Toronto, Ontario, Canada; University of Manchester, United Kingdom

## Abstract

In [^18^F]-FEPPA positron emission topography (PET) imaging, automatic blood sampling system (ABSS) is currently the gold standard to obtain the blood time activity curve (TAC) required to extract the input function (IF). Here, we compare the performance of two image-based methods of IF extraction to the ABSS gold standard method for the quantification of translocator protein (TSPO) in the human brain. The IFs were obtained from a direct delineation of the internal carotid signal (CS) and a new concept of independent component analysis (ICA). PET scans were obtained from 18 healthy volunteers. The estimated total distribution volume (V_T_) by CS-IF and ICA-IF were compared to the reference V_T_ obtained by ABSS-IF in the frontal and temporal cortex, cerebellum, striatum and thalamus regions. The V_T_ values estimated using ICA-IF were more reliable than CS-IF for all brain regions. Specifically, the slope regression in the frontal cortex with ICA-IF was r^2^ = 0.91 (*p*<0.05), and r^2^ = 0.71 (*p*<0.05) using CS-IF.

## Introduction

Neuroinflammation has been implicated to play a role in the pathogenesis of various conditions including Alzheimer’s disease [1], stroke [2], Parkinson’s disease [3], [4] and epilepsy [5]. In general, neuroinflammation is characterized by an overexpression of the translocator protein 18 kDa (TSPO) in activated microglia [6]. Using radioligands that bind to TSPO, positron emission tomography (PET) is able to measure neuroinflammation in the human brain *in vivo* by quantifying TSPO density. The most commonly used radioligand over the last decade has been [^11^C] (R)-PK 11195 [7]. However, [^11^C] (R)-PK 11195 has several disadvantages including low penetration into brain tissue and poor specificity [8]. These limitations triggered interest for the development of new radioligands to overcome the issues above, such as [^18^F]-FEPPA [9], [^11^C]-PBR28 [10], [^18^F]-PBR06 [11], [^18^F]-PBR111 [12], [^11^C]-DAA1106 [13], [^11^C]-DPA-713 [14], and [^11^C]-AC-5216 [15]. Of particular interest, [^18^F]-FEPPA rapidly penetrates brain tissue and has a high affinity and selectivity to TSPO [16] and is, therefore, a favorable radiotracer to quantify TSPO in the human brain.

The quantification of TSPO in dynamic PET data requires an input function (IF) that estimates the arterial plasma radioactivity. For [^18^F]-FEPPA PET imaging, an automatic blood sampling system (ABSS) is currently the gold standard to obtain the blood time activity curve (TAC) required to extract the IF. The blood sampling for [^18^F]-FEPPA PET analysis is often taken at a continuous rate of 2.5 mL/min for the first 22.5 minutes. The main advantage of ABSS is the accurate peak-detection of the IF, which is subsequently used in the kinetic modeling. However, the large volume of blood sampled and the arterial catheterization could induce a physiological effect, infection and/or occlusion [17]. Moreover, ABSS is a costly and time-consuming protocol. Over the years, many researchers have proposed alternative methods to derive the IF in order to minimize the need for repeated blood sampling. One technique extracts the IF directly from PET image data in order to estimate the whole-blood and plasma TACs. Using this method, investigators have successfully obtained the IF from image sources such as the left ventricle to estimate myocardial metabolic rate of glucose (MRGlu) [18], [19] and femoral arteries data from PET imaging to estimate perfusion index in the femoral muscle [20]. In the human brain, the internal carotid artery signal (CS) has been used to extract blood TAC [21], [22]. However, the poor spatial resolution of PET relative to the size of the artery and motion effect which is mostly not easily addressed causes estimation errors [23]. Alternatively, the IF can be obtained from a blind-source separation technique, such as independent component analysis (ICA) [24]. This objective method identifies cranial blood pools and extracts the blood activity signal without the need for manual segmentation of arteries. Further, ICA decreases the noise effect in the blood activity by keeping the principal component and minimizes the noise component corresponding to the lowest eigenvalues (for review, see [24]). Importantly, the success of the ICA approach largely depends on tracer characteristics such as kinetics, washout, tissue distribution, and metabolite formation. Thus, each radiotracer may require an optimized method for ICA. For example, the EPICA algorithm has been demonstrated to accurately describe the FDG tracer in PET imaging [24]. Given the unique tracer kinetics of [^18^F]-FEPPA, the tracer is very likely to require an optimized ICA algorithm.

Although a few studies with [11C]-(R)-PK11195 has employed reference tissue model thanks to its low affinity [25], [26] no suitable reference region with negligible binding has been identified for the high affinity 2nd generation TSPO radiotracers such as [^18^F]-FEPPA. Therefore arterial IF must be obtained to quantify receptor binding. Two common analytic approaches are the graphical plot [27] and compartment model. Our group has previously shown that the two-tissue compartment model (2-TCM) accurately describes the [^18^F]-FEPPA tissue TACs and produces a total distribution volume (V_T_) estimation [16]. In this previous work, ABSS together with manual arterial blood samples were used to generate the IF. In the current study, we aim to find an alternative to ABSS in [^18^F]-FEPPA PET imaging for reasons stated above, namely practicality. For this purpose, we compare the performance of the ABSS with two methods of IF extraction; a) direct delineation of the CS and b) a modified ICA algorithm. We introduce a new concept for the ICA algorithm based on an Asymmetric Laplace distribution (ALD) [28] to improve compatibility with blood region activity features. To test for the interchangeability of the two methods with the ABSS gold standard, we first compared the shape and the area under curve (AUC) of the IFs generated by each method. Subsequently, the linear regression analysis and the Bland-Altman plot were used to test the interchangeability and reliability of the V_T_ estimation given by each method. To further support our modified ICA method for [^18^F]-FEPPA modelling, the proposed ALDICA algorithm is compared to two other known, published ICA methods; EPICA and GGD-ICA. In order to demonstrate the practical applications of the proposed approaches, the estimated V_T_ of each method was compared group-wise between subjects categorized as mixed affinity binders (MABs) and high affinity binders (HABs) [29], [30].

## Materials and Methods

### 1 Human subjects

Eighteen healthy volunteers (7 males, 11 females; aged 24 to 73 years) completed this study. Participants were classified into two groups according to their TSPO polymorphism genotyping (see section below). Nine subjects were classified as MABs and nine subjects as HABs. All procedures were fully explained and subjects provided written informed consent and were approved by the Centre for Addiction and Mental Health Ethics Review Board and conformed to the *Declaration of Helsinke*.

### 2 Polymorphism genotyping

Genomic DNA was obtained from peripheral leukocytes using high salt extraction methods [31]. The polymorphism rs6971 was genotyped variously using a TaqMan assay on demand C_2512465_20 (AppliedBiosystems, CA, USA). The allele T147 was linked to Vic and the allele A147 was linked FAM. PCR reactions were performed in a 96-well microtiter-plate on a GeneAmp PCR System 9700 (Applied Biosystems, CA, USA). After PCR amplification, endpoint plate read and allele calling was performed using an ABI 7900 HT (Applied Biosystems, CA, USA) and the corresponding SDS software (v2.2.2). Individuals with genotype Ala147/Ala147 were classified as high affinity binders (HAB), Ala147/Thr147 as mixed affinity binders (MAB), and Thr147/Thr147 as low affinity binders (LAB) [32].

### 3 Positron emission tomography

All experiments were performed on a 3D high-resolution research tomograph (HRRT) PET scanner (CPS/Siemens, Knoxville, TN, USA). The scanner is made of eight planar detector heads containing LSO/LYSO phoswich detectors, with each crystal element measuring 2.2×2.2×10 mm^3^ and produces 207 image planes. The field of view (FOV) measures 25 cm in axial direction and 35 cm in transaxial direction. The HRRT is characterized by an isotropic spatial resolution of ∼2.5 mm in all 3 directions at the center of the FOV [33]. Each subject was scanned for 120 minutes of dynamic acquisitions in list-mode on the HRRT scanner and images were reconstructed into 256×256×207 cubic voxels measuring 1.22×1.22×1.22 mm^3^ as scheduled below using two algorithms: filtered back projection (FBP) [34] and ordered subset expectation maximization with point spread function (OSEM+PSF) reconstruction [35]. The first frames were of variable length depended on the time between the start of acquisition and the signal recorded in the FOV in both reconstruction algorithms. The subsequent FBP reconstructed image frames were defined as 5×30, 1×45, 2×60, 1×90, 1×120, 1×210, and 22×300 seconds. The subsequent OSEM reconstructed image frames were defined as 2×7, 2×8, 3×10, 2×15, 2×30, 1×45, 2×60, 1×90, 1×120, 1×210, and 22×300 seconds.

### 4 Magnetic resonance imaging

Magnetic resonance imaging (MRI) scans were acquired for subjects using a General Electric (Milwaukee, WI, USA) Sigma 1.5 T MRI scanner. The slice thickness measured 2 mm and the repetition time was greater than 5.300 milliseconds. The echo time = 13 milliseconds and the flip angle = 90°. The acquisition matrix measures 256×256, and the FOV measures 22 cm. 2D axial proton density (PD) MRI images were utilized for anatomic delineation of the cerebellum, the frontal and temporal cortex, striatum, and thalamus regions using in-house software, ROMI [36]. A discrete cosine transform (DCT) was used to wrap the standard template of ROIs to each subject.

### 5 Blood sampling

Approximately 185±20 MBq (5±0.5 mCi) of [^18^F]-FEPPA was administered intravenously as a bolus injection. During PET acquisition, the whole-blood radioactivity was continuously measured via a cannulation to the radial artery and using ABSS (Model #PBS-101 from Veenstra Instruments, Joure, Netherlands) at a rate of 2.5 ml/min for the first 22.5 min. Manual arterial blood samples of 4–10 ml were taken at 1.5, 2.5, 7, 15, 30, 45, 60, and 90 minutes in order to assess the concentration ratio of radioactivity in whole blood to the plasma and to calculate the metabolite composition in the plasma. The blood-to-plasma ratio was fitted by a bi-exponential. Plasma composition was determined using Hilton method [37] and the fraction of the un-metabolized radioligand was fitted by a Hill function 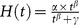 in order to correct plasma TACs for metabolite. The correction for delay and dispersion of the radioligand was performed as follows: the delay between the manual plasma activity and the activity recorded over 50 seconds in the FOV was fitted to an irreversible 1-compartment model with whole-blood activity as an input function. The dispersion effect in the ABSS line was modeled as 
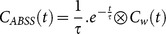
 [38], where 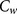 is the concentration of tracer in the whole-blood at time *t* and τ as taken equal to 16 seconds. The ABSS whole-blood radioactivity corrected for the dispersion and delay effects was used to build the input function as follows:



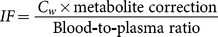
(1)


The tissue TACs were generated using in-house software ROMI. A standard template of ROIs was fitted to each subject’s MRI. The MRI was co-registered (SPM2, Department of Cognitive Neurology, London) to a time-average of the dynamic PET images and the transformation was subsequently applied to the ROI map. TACs were extracted from each ROI placed in the dynamic PET images.

### 6 Internal carotid signal delineation

**Figure 1:**
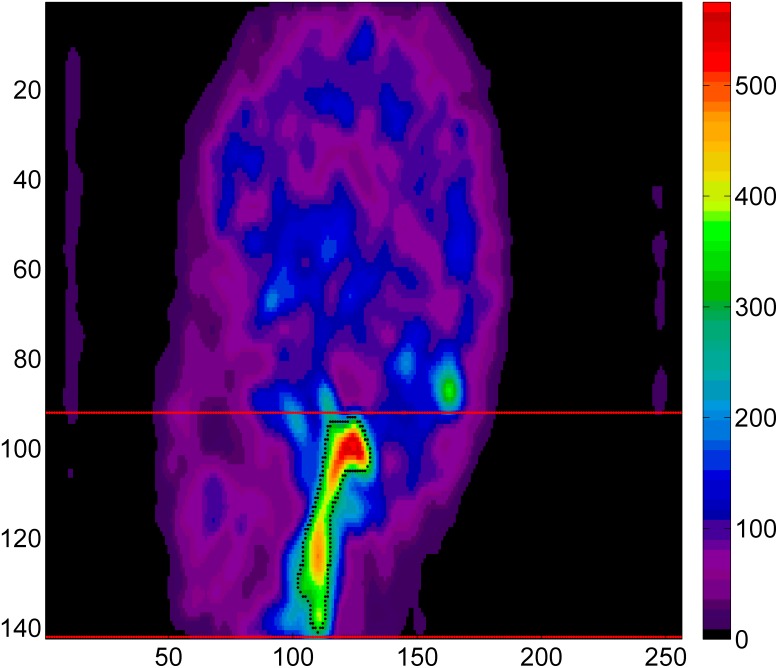
Internal carotid segmentation performed on OSEM-PSF images using automatic thresholding for one subject. The red lines illustrate the lowest 36 planes containing the internal carotid artery. The black line surrounding the carotid artery represents the automatic binary mask.

The carotid ROIs were automatically delineated over the PET images. The binary masks were created by thresholding the mean image of the first 10 frames (2 minutes of scan) at 0.48×maximum intensity in which the optimal shape of the carotid artery was clearly displayed in the image series (N.B. The threshold has been determined empirically). For [^18^F]-FEPPA, the whole-blood TAC was extracted from the carotid ROIs as a mean of the ten highest pixel activities per plane, as explained elsewhere [22]. Only those voxels located within the lowest 36 planes (43.9 mm) were considered as carotid signal (Fig. 1).

### 7 Independent component analysis

**Figure 2:**
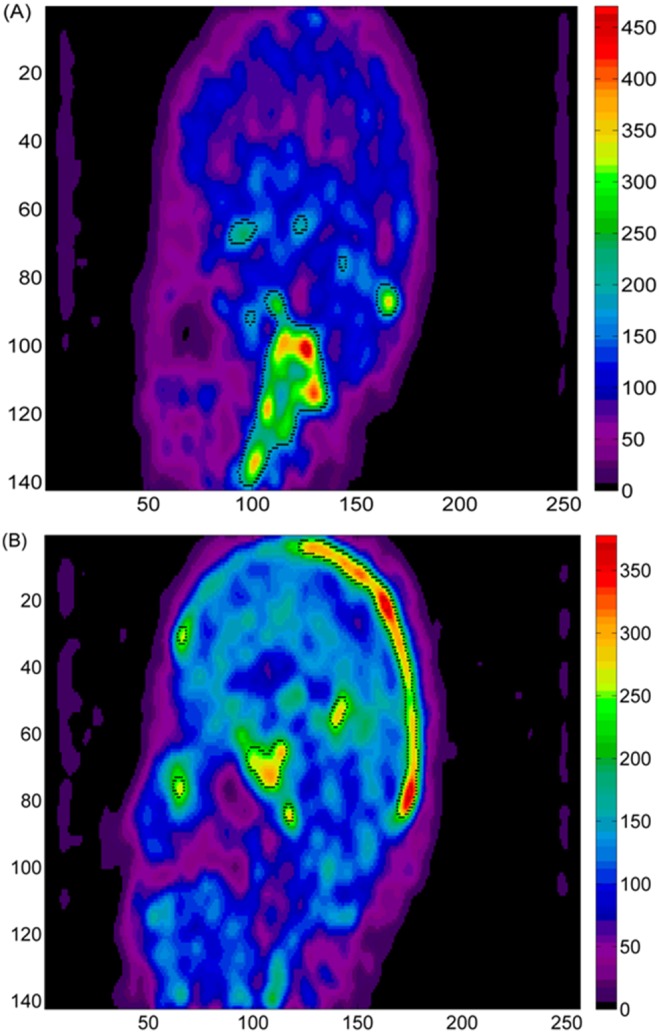
ROIs extracted for the ICA algorithm for the same subject in Fig. 1. Binary masks were created from the time-averaged image (first 10 frames) and applied onto each plane to automatically select the whole-brain blood regions over all planes. A) depict the arterial activity. B) depict the superior sagittal sinus.

The same intensity threshold (0.48×maximum) was used to segment blood region ROIs of the brain as described in the CS methodology. The blood ROIs were applied to include the whole vascular system in brain constitute of arterial and venous activity for the ICA algorithm in order to enhance the presence of two different spatial distributions. Additionally, a 3D cubic Gaussian filter with FWHM = 3 mm was used to smooth activity within the blood ROIs (Fig. 2).

**Figure 3:**
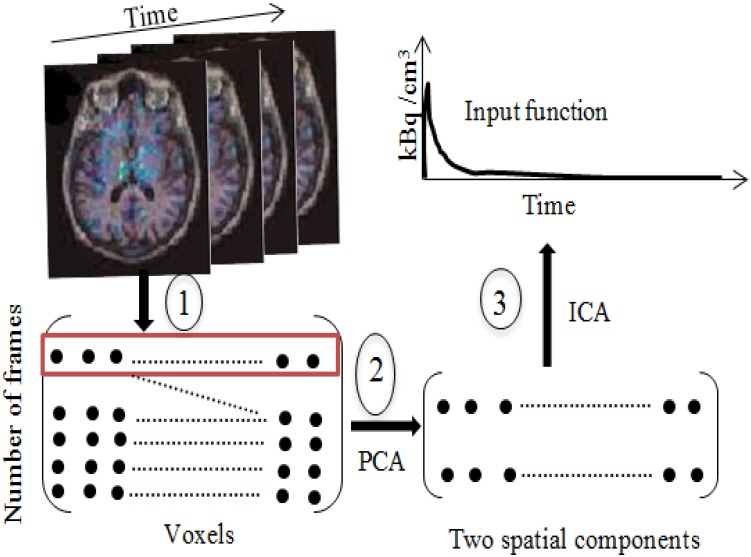
Illustration of the ICA algorithm process steps. (1) the dynamic PET images derived from sequential measurement of the radioactivity are re-arranged into 2D matrix, the first dimension refers to time acquisition fames and the second dimension refers to the spatial distribution. (2) the principal component analysis reduce matrix dimension in order to keep the most significant activity (if columns of a mixture have relatively similar TACs, then, the corresponding columns tends to be estimated as one components). (3) update the de-mixing matrix until found convergence in separation between the two components.

Masks were applied to the rest of the dynamic image series to select the time activity of voxels within the blood ROIs. The 4D data were rearranged into a 2D matrix 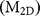. The first dimension 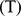 is the time course and the second dimension 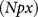 is the spatial distribution of pixels in each ROI. Specifically, arterial and venous activity was largely identified in addition to the undesired signal. The dimension of 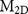 was reduced from 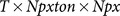 (where 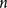 is the number of independent components of interest *e.g.* arterial and venous activity) using the principal component analysis algorithm. This technique determines the Eigenvectors 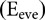 and Eigenvalues 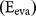 of the covariance matrix 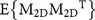 and uses them as a basis for the reduction. A flow diagram of the overall process is illustrated (Fig. 3). Specifically, the matrix of the mixture of the n independent sources 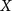 was computed as a product 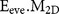 and used as the input data in the ICA algorithm [39].

The matrix 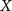 is then defined as:
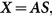
(2)where the matrices 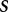 and 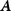 are unknown and X is the mixed activity. The ICA algorithm allows for estimation of 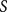 through a de-mixing matrix 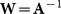. Hence, the source matrix 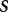 is approximated by an estimate 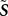 given by:



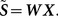
(3)In this work, we used the maximum likelihood estimation to determine 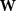. For this purpose, we defined the negative of the log likelihood as a cost function, as defined by the Amiri algorithm [40]:
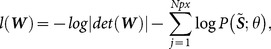
(4)where 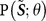 represents the distribution of the sources. Further detail on the calculation of 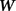 and the ADL parameters estimation are given in Appendix A and B in S1 File. A Summarize of ICA algorithm is given in appendix C in File S1.

### 8 Whole-blood calibration and input function generation

The whole-blood curves extracted from the ROIs by CS and ICA were calibrated to the ABSS-IF. Both curves were calibrated with one manual arterial blood sample taken at 15 minutes post-injection. In addition, a single inversion point at 1.5 minutes post-injection generated by the algorithm was corrected using one blood sample. The IFs were calculated using the derived whole-blood curves and the Hill function and bi-exponential function. The parameters of the two functions were calculated using an in-house software (Pharmacokinetic Estimation for Radioligand Images (P.E.L.I)) which gives the possibility to model manual samples with different pre-defined function such as linear interpolation, mono-exponential, bi-exponential and Hill functions. Unlike ABSS-IF, the IDIFs were free from delay and dispersion effects.

### 9 Kinetic modelling

The total V_T_ which represents the ratio at the equilibrium of the concentration of radioligand in tissue to that in plasma (*i.e.* including the specific binding, nonspecific binding and free radioligand in tissue) was assessed. For the 2-TCM, V_T_ was defined as 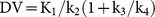 where 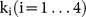 were the independent variables estimated by the compartmental model, as described elsewhere [41]. The weighted nonlinear fitting was performed with the Levenberg-Marquardt algorithm using an implemented trust-region. The cerebral blood volume was assumed in the trust-region function as 5% of the gray matter tissue [42]. The fitting curve and the brain data for each frame were weighted to the other frames using the trues 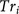 in the FOV, where *i* is the frame index as described by:
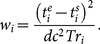
(5)where 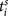 and 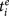 are the start and end time of the frame, respectively, and 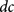 is the decay correction coefficient given by: 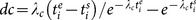 with 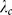 = 6.3×10^−2^ per minute, that is the decay of ^18^F.

### 10 Statistical analysis

The IFs derived by CS and ICA were compared to the ABSS-IF using the AUC ratio. Pearson correlation coefficients were used to evaluate the strength of the relationship between CS-IF, ICA-IF and ABSS-IF. Bland-Altman plot were performed to test the interchangeability of the V_T_ calculated by different methods in the temporal and frontal cortex, cerebellum, striatum, and the thalamus. The regression coefficients were tested using a one-sample t-test with statistical significance set to a value of p<0.05. Repeated-measures ANOVA (RMANOVA) was performed using V_T_ values calculated using the IF from each method and according to their genotype (i.e. HAB and MAB subjects).

## Results

### 1. Input Functions

**Figure 4:**
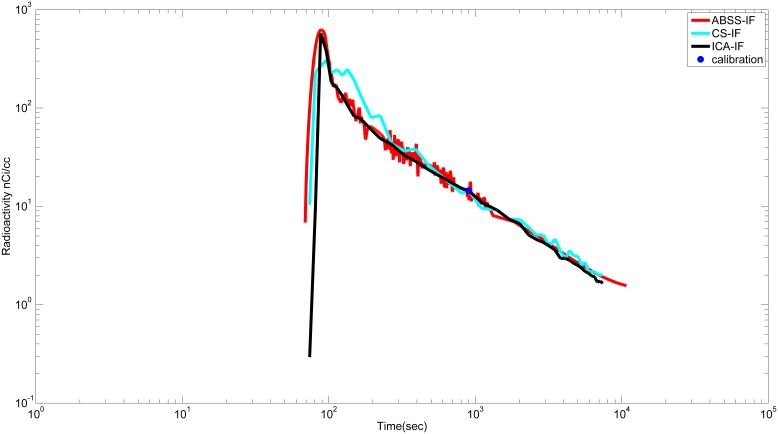
A typical double logarithmic scale of the input function estimated by CS and ICA plotted against the ABSS-IF. Two arterial blood samples were used to correct for a small inversion at 1.5 minutes (green point) and to calibrate curves at 15 minutes post injection (blue point).

The ABSS-IFs, CS-IF and ICA-IF curves demonstrated a substantial similarity in terms of shape for each individual subject. The peak activity of the CS-IF was reached at the same time as the ABSS-IF curve, but was notably smaller in amplitude. In contrast, the peak of the ICA-IF curve was reached slightly later (∼5 sec) and was much sharper than the ABSS-IF curve. Over the course of 15 minutes, the ABSS-IF and the two image-derived curves were similar (see Fig. 4).

Within each individual, the peak (blood activity in the first 180 seconds) and the tail (blood activity in the remaining time of scan) AUCs were calculated for the ABSS-IF, CS-IF and ICA-IF curves. The AUCs (mean ± SD) for the CS-IF peak were underestimated compared to the AUCs of the ABSS-IF peak (ratio = 0.74±0.12). In contrast, the AUCs for ICA-IF peak were overestimated compared to the AUCs of the ABSS-IF peak (ratio = 1.07±0.18). However, the ICA-IF and ABSS-IF tail AUCs ratio were very similar (ratio 1.03±0.10), whereas the CS-IF and ABSS-IF tail AUCs ratio were slightly overestimated (ratio = 1.12±0.14).

### 2. Reliability of V_T_ Estimations

#### 2.1 Group-wise comparisons between HAB vs. MAB

**Figure 5:**
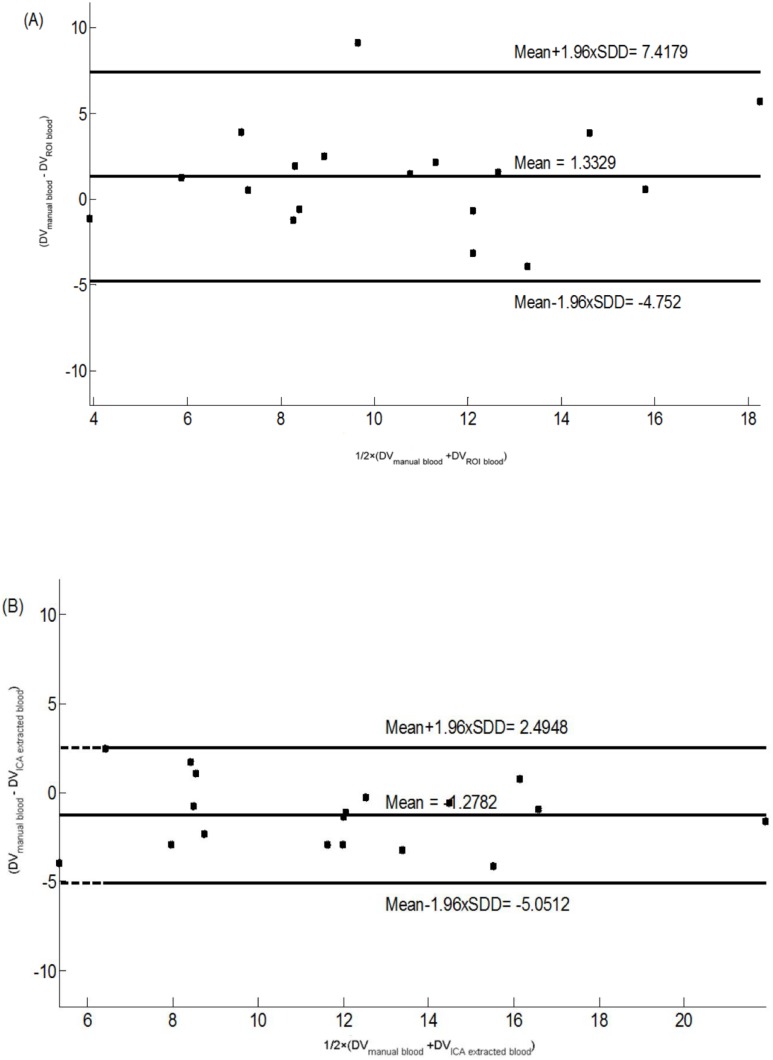
Bland Altman plot of total distribution volume (V_T_) in the frontal cortex comparing (A) ABSS-IF versus CS-IF, and (B) ABSS-IF versus ICA-IF. All candidates were within the 95% of limits agreed for 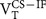 and 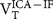, with the exception of one subject for 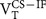.

A group comparison for the frontal cortex is depicted in Fig. 5 (for the cerebellum, temporal cortex, striatum and thalamus see S1.1, S1.2, S1.3, and S1.4 Figs in S2 File). Within each group, the RMANOVA revealed no significant difference in V_T_ estimations from the ICA, CS and ABSS methods for HABs (F (2, 16) = 0.2, p = 0.82) and MABs (F (2, 16) = 1.22, p = 0.32), respectively.

#### 2.2 *Linear Regression*

We first conducted a simple linear regression analysis of the data (n = 18) for frontal and temporal cortex, cerebellum, striatum and thalamus regions. Results of regression analysis comparing V_T_s calculated using ICA-IF and V_T_ calculated using ABSS-IF are shown in Table 1. Overall, V_T_ using ICA-IF provided a more reliable estimation of gold standard V_T_ using ABSS-IF (r^2^> = 0.88) than V_T_ using CS-IF (r^2^<0.88).

**Table 1:** Results based on Regression Analysis V_T_ calculated using CS-IF versus using ABSS-IF and V_T_ calculated using ICA-IF versus using ABSS-IF.

	V_T_ calculated using CS-IF *vs* V_T_ using ABSS-IF					V_T_ calculated using ICA-IF *vs* V_T_ using ABSS-IF				
	r^2^	b_0_	b_1_	*t*	*p*	r^2^	b_0_	b_1_	*t*	*p*
Frontal cortex	0.71	1.13	0.85	4.01	0.0009	0.91	0.03	1.09	6.04	1.1E-04
Temporal cortex	0.70	0.34	0.95	5.19	8.8E-05	0.88	2.24	1.14	3.21	0.0053
Striatum	0.78	0.04	1.01	5.5	4.0E-05	0.89	1.64	1.05	3.74	0.0017
Thalamus	0.87	1.76	0.83	3.52	0.0027	0.92	-0.43	1.17	7.54	1.1E-06
Cerebellum	0.84	1.17	0.84	3.60	0.0023	0.90	0.98	1.01	4.37	0.0004

r^2^: coefficient of determination, b_0_: Intercept, b_1_: Slope, *t*: t-test value, *p*: p-value.

#### 2.3 Bland Altman plot

**Figure 6:**
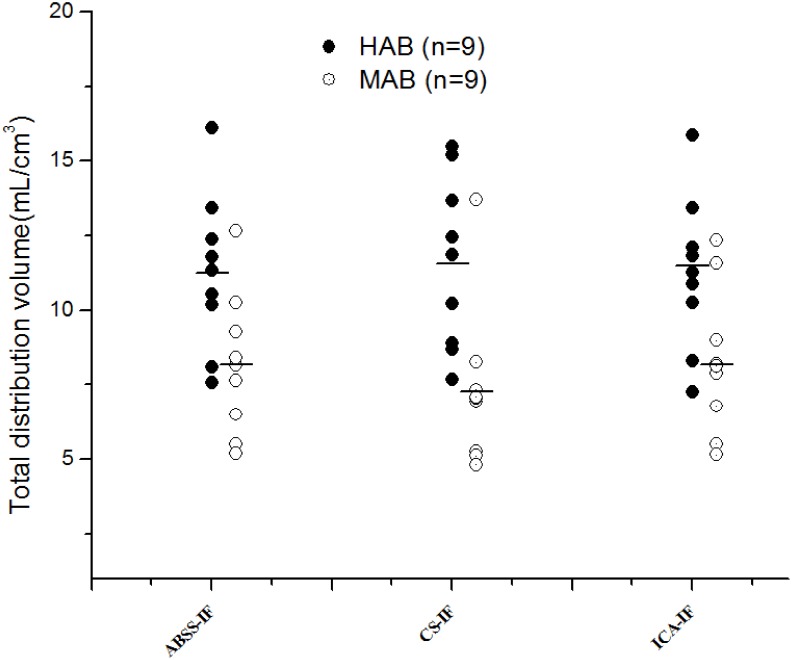
Group comparison of total distribution volume (V_T_) in the frontal cortex for high affinity binders (HABs) and mixed affinity binders (MABs) calculated respectively with ABSS-IF, CS-IF and ICA-IF.

The bias of the measurements between V_T_ using ABSS-IF 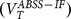 and V_T_ using CS-IF 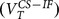 calculated in the frontal cortex was 1.33 mL/cm^3^ and the width of 95% of limits agreement was 12.17 mL/cm^3^ containing 17/18 of difference scores (Fig. 6A) (for the cerebellum, temporal cortex, striatum, and thalamus see S2.1A, S2.2A, S2.3A, and S2.4A Figs, respectively, in S2 File). In contrast, the bias of the measurements between 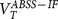 and V_T_ using ICA-IF 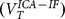 in the same region was −1.27 mL/cm^3^ and the width of 95% of limits agreement was 7.54 mL/cm^3^ containing all difference scores (18/18) (Fig. 6B) (for the cerebellum, the temporal cortex, the striatum, and the thalamus (see S2.1B, S2.2B, S2.3B, and S2.4B Figs, respectively, in File S2). Therefore, the magnitude of bias was smaller in 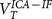 than 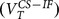.

## Discussion

In this study, we developed a new algorithm for the ICA method to extract the IF necessary for TSPO quantification in addition to the CS method used in other studies [22]. The extracted TACs from PET data include signals from both the parent and the metabolized radiotracers. Therefore, the blood TACs extracted from PET data need to be corrected to the blood-to-plasma ratio and, parent fraction of the radiotracer, which can be achieved by calibrating with one arterial or venous sample [18], [22], [43]. In this work, the whole-blood-to-plasma ratio was determined by fitting manual sample by a bi-exponential function, and the un-metabolized [^18^F]-FEPPA activity was modeled by a Hill function because of the fast metabolism of this tracer as previously demonstrated [9], [16].

The CS method was based on the segmentation of the carotid to extract blood activity. However, the identification of the internal carotid arteries requires high-resolution dynamic PET images or a co-registration with the subject MRI [23]. Due to the small size of the carotid and its elasticity (*i.e.* the artery can be stretched or bent during the MRI or PET scans), co-registration leads to an alignment error which requires further correction using a simple rigid body co-registration [23]. Therefore, the segmentation of the carotid in this study was carried out directly on OSEM+PSF [^18^F]-FEPPA PET which has been demonstrated to be robust against the partial volume effect [44]. The left and the right internal carotid were easily recognized in the early summed frames of a dynamic PET scan. Then, only a number of highest pixels (ten highest pixels) per plan were selected within the carotid ROIs to minimize the spill-in and spill-out effects. The number of pixels was established by experiment as performed elsewhere [22] and according to the spatial resolution of the scanner (in [22], Mourik et al used four highest pixels for [11C]-flumazenil PET data acquired using an ECAT EXACT HR+ scanner with spatial resolution = 4.3 mm at the center of FOV). The CS-IFs obtained are marginally compared to the ABSS-IFs and the AUCs ratio between CS-IF and the gold standard IF is low for the peak and high for the tail. In addition the inter-subject variation is high for both peak and tail (0.12 and 0.14 respectively) although the scaling of the IF by a manual arterial sample to compensate the underestimation as suggested by Zanotti-Fregonara et al [23]. Despite the fact that the use of the highest pixels is simple, largely automatic and fully reproducible; this method is very sensitive to the motion effect and depends of the number of the selected high intensity volumes. Given that pixels ROI was defined on the average image over the two first minutes of scan, participant may move from its first location and, thus, leads to an inaccuracy in the blood TAC. Moreover, the tail of the IF plays an important role in VT variation when scans are long (120 to 180 minutes) and the metabolism is fast as in the case of FEPPA PET. As a result, the agreement between estimated V_T_s using CS-IF and ABSS-IF was only marginally significant (0.70≤ r2≤0.87) and the Bland Altman plot showed qualitative difference between 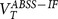 and 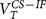 which appear as a wide width of agreement limits (12.17 mL/cm^3^). The source of this discrepancy includes potential contributors such as patient movement during the scans and the remaining partial volume effect, although it has been reduced by OSEM+PSF reconstruction algorithm.

The ICA method was used to derive the IF directly through a source signal de-mixing process. Unlike deterministic blind source separation (BSS) such as the non-negative matrix factorization (NMF), the ICA is more flexible and, therefore, able to model initial sources by different probabilistic distributions that accurately adapt to physical and physiological characteristics. The ICA algorithm was applied on *a priori* determined automatic anatomical segmentation of the brain vessel system. The advantage of collecting the activity from regions where the radiotracer activity in the blood is highly recognizable is to increase the number of samples and satisfy the Central Limit Theorem [43]. The segmentation was performed on PET data reconstructed with OSEM algorithm where the number of subsets = 6 and the number of iterations = 12, allowing for clear recognition of the high signal contrast of cerebral vessel activity to background noise. In doing so, however, there was some loss in precision of the tissue time activity. Especially, the application of ICA on the carotid region ROIs, fails to extract the IF. This is mainly due to the fact that the statistical property of mixture in segmented ROIs is violated (at least two distinct independent distributions built the mixture). Indeed, the segmented carotid ROIs is a mixture of arterial activity distribution and a random background noise distribution which led to the extraction of noisy curves (see.S3 Fig. in S2 File). In contrast, the activity within the whole-brain ROIs was assumed to be mixture of radiotracer activity in venous and the artery. The spatial distributions of these sources were slightly different because of several reasons. 1) the field of view of the scanner covers more voxels in venous than artery vessels; 2) the size of the venous cerebral vessels is large compared to the arterial ones which cause less partial volume error; 3) and likely due to the tracer binding to the vessel walls (i.e. the endothelium and smooth wall muscles).

Previously, researchers have used several different measures to calculate the independence from the mixed signal (*i.e.* kurtosis, entropy, mutual information, and the likelihood) [43]. In this study, we modeled the spatial distribution of blood activity through the PET acquisition time by an ALD. The choice of this distribution is supported by the fact that the tracer is uniformly distributed in the venous vessels and arterial ones. In terms of voxel intensity, the histogram of the spatial distribution of both components was close to a Laplace distribution, which can be skewed to the left or right. The ALD was flexible through its skew parameter to fit this distribution (*i.e.* The two components were extracted as a first and a second independent source relative to the first and second principal components correspond to the highest eigenvalue). The first source closely resembled the ABSS-IF according to the peaked time and was visually identified as the desired arterial blood activity. It was subsequently used to generate the IF.

**Figure 7:**
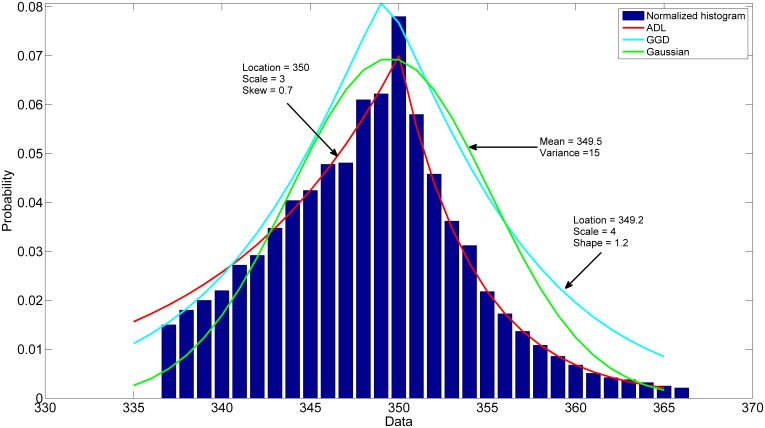
Illustration of the normalized histogram of the first source, ADL, GGD, and Gaussian distributions respectively. The plot describes the data fit by three different distributions. The Gaussian distribution does not show a good model to represent data. The GGD fit better the sharper features of the histogram. However, it fails to fit well the asymmetry of data. The ADL is more appropriate to model the sharper feature of the histogram and, moreover, follows the asymmetric distribution of data through its skew parameter.

In further support of the ALDICA algorithm for [^18^F]-FEPPA PET data, it was compared to a non-parametric ICA based on the fast fixed-point EPICA algorithm, explained in detail elsewhere [24] (source code is freely available at http://home.att.ne.jp/lemon/mikan/EPICA.html), and a parametric ICA based on two distributions; a) ICA based on a Gaussian cost function and; b) an ICA based on maximum likelihood estimate and the general Gaussian distribution (GGD) [39]. The EPICA algorithm successfully extracts TAC from FDG PET data [23]. In case of [^18^F]-FEPPA PET data, however, this algorithm failed to extract the IF for several reasons. In the case of FDG PET, the blood-tissue histogram differentiates blood and tissue signal components and EPICA successfully emphasizes each component independently. In [18F]-FEPPA, however, this no longer the case as the histogram consists of two close distributions; venous and arterial blood signal in selected ROIs. EPICA failed in the separation of the two distributions in the selected ROIs because of their similarity with the applied cost function. The EPICA method works well with FDG due to its equal distribution in brain tissues, resulting in very similar tissue TAC scales and can be estimated as one component. In contrast, [^18^F]-FEPPA is not as equally distributed across tissues and hence, is difficult to separate blood from different tissue TAC scales. In the standardization process, the EPICA algorithm increased undesired signal (e.g. tissue component and noise). Moreover, the parameters of the cost function are fixed in the equation by the user and not by estimation from the data. For FDG PET data, as shown by Naganawa et al (2005), changing these parameters within a specified range (10< λ <100; 0.1< m<0.5) did not influence the result. However, the parameters λ and m applied to [^18^F]-FEPPA PET data revealed a dependency on the selected values for the parameters. Further, we compared the ALD to other parametric spatial distribution models which, in general, are more flexible with manipulation of the data distribution. However, the Gaussian distribution was unappropriated to extract independent components because of the assumption made on the mixture (the mixture of independent components is more Gaussian than the independent components [43]). The GGD, in contrast, is more adaptable through its third parameter (shape parameter (SP)) and covers many distributions (SP<2, super-Gaussian distribution; SP = 2, Gaussian distribution; SP>2, sub-Gaussian). The asymmetric distribution of ALDICA function allows for a good fit to the data compared to EPICA and the parametric models, as highlighted in Fig. 7. In summary, these comparisons support ALDICA as the spatial distribution function of choice in dynamic [^18^F]-FEPPA imaging.

The obtained IFs, using ADLICA were similar to the ABSS-IF in shape, but not in magnitude. This was due to two ambiguities in the ICA algorithm: magnitude and permutation. To solve the magnitude issue, one manual blood sample was used to scale the extracted IFs at 15 minutes post-injection to calibrate the peak and the tail. The post-injection sample at 15 minutes was chosen due to the fact that, for [^18^F]-FEPPA, this represents the near end of the tracer delivery to the tissue and the beginning of the blood clearance. Hence, at this time, the used plasma sample might be more accurate for calibration. The identification of the first and the second extracted components as arterial blood activity and venous or tissue activity was a sensitive task due to the permutation ambiguity of ICA. For the majority of the subjects (14/18), the magnitude of the second component, inspected visually, was higher than the first and peaked approximately 5 seconds later than the first component, which represent venous activity for reasons cited above (see.S4 Fig. in S2 File). For the remaining subjects (4/18), the second component appeared as a tissue activity shape which is explained by strong signal contamination of the blood region by surrounding tissue activity. In addition, for 2 of 18 subjects, the arterial blood activity was identified as the second component. Finally, a small single inversion point in the curve was created by the algorithm at ∼1.5 minutes following tracer injection. Importantly, this inversion did not affect the estimated total VT values using IF. After correcting for calibration concerns, the peak AUCs and the tail AUCs calculated with ICA represent an promising estimation compared to those obtained with ABSS (peak ratio = 1.07±0.18, tail ratio = 1.03±0.10). These estimations fall within an acceptable range of variation. Notably, the inter-subject variation is largely due to the threshold used to create the ROIs. We chose to keep this threshold constant (48% of maximum intensity) across all subjects. However, it is possible to reduce the variance by selecting a subject-specific threshold. In addition, the inter-frame motion is a hidden issue which is difficult to correct and contribute to the high variation of the tail ratios. In order to determine if the slight difference in peak activity timing between ICA-IF and ABSS-IF curves, we shifted the ICA-IF curve to the left to match the peaks. There was a negligible influence on the estimated V^T^ (∼1% difference between V^T^ estimated with ICA-IF and the shifted ICA-IF). Moreover, V_T_ estimations obtained with ICA-IF were highly correlated (0.88≤ r^2^≤0.92) with ABSS-IF. The RMANOVA revealed no significant difference between 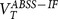 and 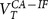 Further, the Bland-Altman plot revealed only a negligible difference between 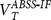 and 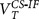 which appear as a small width of agreement limits (7.54 mL/cm^3^).

Although the possibility of avoiding the use of ABSS to extract IF from PET data, arterial samples are usually needed to calculate the blood-to-plasma ratio and the un-metabolized radioligand. This issue should be addressed in further studies either by venous substitute [44] or by population base correction. Two other issues worthy to be addressed in this work. First the visual identification of the arterial blood activity and second the manual correction of the small inversion. Finally, a new methodology using clustering to extract a grey matter tissue reference region should be subject to future studies to quantify microglia activation using [^18^F]-FEPPA as applied to [11C]-(R)-PK11195 tracer [45].

## Conclusion

This study supports ICA from OSEM [^18^F]-FEPPA PET as an interchangeable method to the gold standard ABSS to quantify TSPO in the human brain. The CS method produces slightly less accurate V_T_ and may, therefore, not be a reliable candidate as an alternative method to ABSS. The major advantage of these techniques is the extraction of IFs with low blood sampling and rapid processing time. Both ICA and CS methods were performed solely on PET image data, eliminating the need for MRI-based segmentation of blood regions. However, manual blood sampling was still required to calculate the metabolite and blood-to-plasma ratio, in addition to calibration. This limitation needs to be improved with future work.

## Supporting Information

S1 File **Appendix A, B and C.**
(DOCX)

S2 File **This file contains the supporting information figures.** S1.1 Fig: Direct comparison of total distribution volume (V_T_) in the cerebellum for high affinity binders (HABs) and mixed affinity binders (MABs) calculated respectively with ABSS-IF, CS-IF and ICA-IF. S1.2 Fig: Direct comparison of total distribution volume (V_T_) in the temporal cortex for high affinity binders (HABs) and mixed affinity binders (MABs) calculated respectively with ABSS-IF, CS-IF and ICA-IF. S1.3 Fig: Direct comparison of total distribution volume (V_T_) in the striatum for high affinity binders (HABs) and mixed affinity binders (MABs) calculated respectively with ABSS-IF, CS-IF and ICA-IF. S1.4 Fig: Direct comparison of total distribution volume (V_T_) in the thalamus for high affinity binders (HABs) and mixed affinity binders (MABs) calculated respectively with ABSS-IF, CS-IF and ICA-IF. S2.1 Fig: Bland-Altman plot of total distribution volume (V_T_) in cerebellum region. S2.2 Fig: Bland-Altman plot of total distribution volume (V_T_) in temporal cortex region. S2.3 Fig: Bland-Altman plot of total distribution volume (V_T_) in striatum region. S2.4 Fig: Bland-Altman plot of total distribution volume (V_T_) in thalamus region. S3 Fig: Log-Log scale plot of ABSS-IF and ICA-IF performed on carotid region. S4 Fig: Log-Log scale plot of the first and the second independent components.
(DOC)
