# Supplementary material for: Image Derived Input Function for [18F]-FEPPA: Application to Quantify Translocator Protein (18 kDa) in the Human Brain
Source: PLoS One. 2014 Dec 30;9(12):e115768. doi: 10.1371/journal.pone.0115768 (PMC4280118; doi:10.1371/journal.pone.0115768)
Supplement: S1 File — Appendix A, B and C. (DOCX) [file pone.0115768.s001.docx]

**File S1: Appendix**

1. **De-mixing matrix update**

The de-mixing matrix at iteration $t+1$is estimated from the preceding $W$estimation using the steepest descent gradient [37] as follows:

$W\left(t+1\right)= W\left(t\right)-\eta_{t}\left(I+\Phi\left(\tilde{S}\right)\tilde{S}^{T}\right)W\left(t\right),\text{ (4)}$With

$\Phi\left(\tilde{S}\right)=\left[\text{φ}\left(\tilde{\text{S}}_{1}\right),\text{ φ}\left(\tilde{\text{S}}_{2}\right)\right]\text{ }^{\text{T}}\text{ (5)}$and

$\text{φ}\left(\widetilde{\text{S}_{\text{j}}}\right)=-\frac{\partial\log P\left(\widetilde{\text{S}_{\text{j}}};\theta_{j}\right)}{\partial\theta_{j}}.\text{ (6)}$The subscript$j$in eq. (7) indicates the jth source. The optimal nonlinear function $\text{ φ}\left(\widetilde{\text{S}_{\text{j}}}\right)$requires the knowledge of the distribution$p\left(\widetilde{\text{S}_{\text{j}}};\text{ θ}_{\text{j}}\right)$In this work, we have modelled $p\left(\widetilde{\text{S}_{\text{j}}};\text{ θ}_{\text{j}}\right)$by an ALD defined by:

$p\left(\widetilde{\text{S}_{\text{j}}};\text{ θ}_{\text{j}}\right)=\frac{\rho_{j}\left(1-\rho_{j}\right)}{\sigma_{j}}\text{e}^{(\frac{-\left(\widetilde{\text{S}_{\text{j}}}-\text{μ}_{\text{j}}\right)}{\text{σ}_{\text{j}}}\left[\text{ ρ}_{\text{j}}-\text{ I(}\hat{\text{S}}_{\text{j}}\leq\text{μ}_{\text{j}}\text{)}\right])}\text{ }(7)$where $\theta_{j}=\left(\text{ μ}_{\text{j}},\text{ }\sigma_{j},\text{ ρ}_{\text{j}}\right)$is the vector of parameters: 0<$\text{ ρ}_{\text{j}}$<1 is a skew parameter, $\sigma_{j}>0$is a scale parameter$-\infty<\text{ μ}_{\text{j}}<+\infty$,is a location parameter, and I(.) is the indication function defined as $I\left(\hat{\text{S}}_{\text{j}}\leq\text{ μ}_{\text{j}}\right)=1\text{ if }\hat{\text{S}}_{\text{j}}\leq\text{ μ}_{\text{j}}\text{ otherwise }I\left(\hat{\text{S}}_{\text{j}}\leq\text{ μ}_{\text{j}}\right)=0)$

1. **Asymmetric Laplace distribution**

The estimation of the ALD parameters was completed using the maximum likelihood estimator as follows (for more details see [24]):

$\hat{\mu}_{j}=argmin_{\mu}\sum_{i=1}^{Npx} \varepsilon_{\hat{\rho}_{j}}\left(\hat{s}_{j,i}-\text{ μ}_{\text{j}}\right)\text{ (8)}$ $\hat{\sigma}_{j}= \frac{1}{Npx}\sum_{i=1}^{Npx} \varepsilon_{\hat{\rho}_{j}}\left(\hat{s}_{j,i}-\hat{\mu}_{j}\right),\text{ (9)}$where $\varepsilon_{\hat{\rho}_{j}}=\left[\hat{\rho}_{j}-\text{I}\left(\hat{s}_{j,i}\leq\hat{\mu}_{j})\right]\right)$.

$\hat{\rho}_{j}= \frac{\hat{a}_{j}+\sqrt{\hat{a}_{j}^{2}-\left(\overline{s_{j,i} }-\hat{\mu}_{j}\right)\hat{a}_{j}}}{\overline{s_{j,i} }-\hat{\mu}_{j}}, \text{ (10)}$where

$$\hat{a}_{j}=\frac{1}{Npx}\sum_{i=1}^{Npx} (s_{j,i}-\hat{\mu}_{j})I\left(s_{j,i}\leq\hat{\mu}_{j}\right).$$

1. **Summarize of ICA algorithm**

Finally, we can summarize our new algorithm starting from the measured data until the separation of the spatial independent components and their IF, by the following steps:

1. Compute the covariance matrix $C=MM^{T}$.

Compute the eigenvalues $\Lambda= diag\left(\lambda_{1} , \lambda_{2} , \lambda_{3}. . . , \lambda_{T} \right)$and the eigenvectors $E = \left(E_{1} ,E _{2},E_{3}, . . .E_{T}\right)$of the covariance matrix. The dimension of each column of $E$is $T.$

1. The eigenvectors form the basis of the data, and the eigenvalues are used to order them according to the variance in the data that they represent. The principal factor matrix columns are composed of the $n < T$eigenvectors$\left[E_{eve}=\text{ }\left(E_{1}, . . . ,E_{n}\right)\right]$, and considered as the temporal components. The square matrix $\left[E_{eva}\text{ } =\text{ }\left(\Lambda_{1}, . . .\text{Λ}_{n} \right)\right],\text{ }$is composed of the $n < T$principal eigenvalues. The principal spatial components are then computed as $X = E_{eve}.M$.
2. Initialize $W,$ randomly using values between 0 and 1.
3. Initialize $\text{θ = }\left(\text{μ = 0, σ = 1, ρ = 0.5}\right).$
4. For current $W,$estimate $\theta$ using eq. (8), (9) and (10).
5. Minimize the cost function according to eq. (3).
6. If the cost function is minimal, terminate, else update $W$according to eq. (4) and go to step (5).
7. Calculate whole blood as $C_{w}= WE_{eve}^{T}$.
8. Calculate IF as
