# Supplementary material for: Image Derived Input Function for [18F]-FEPPA: Application to Quantify Translocator Protein (18 kDa) in the Human Brain
Source: PLoS One. 2014 Dec 30;9(12):e115768. doi: 10.1371/journal.pone.0115768 (PMC4280118; doi:10.1371/journal.pone.0115768)
Supplement: S2 File — This file contains the supporting information figures. S1.1 Fig: Direct comparison of total distribution volume (VT) in the cerebellum for high affinity binders (HABs) and mixed affinity binders (MABs) calculated respectively with ABSS-IF, CS-IF and ICA-IF. S1.2 Fig: Direct comparison of total distribution volume (VT) in the temporal cortex for high affinity binders (HABs) and mixed affinity binders (MABs) calculated respectively with ABSS-IF, CS-IF and ICA-IF. S1.3 Fig: Direct comparison of total distribution volume (VT) in the striatum for high affinity binders (HABs) and mixed affinity binders (MABs) calculated respectively with ABSS-IF, CS-IF and ICA-IF. S1.4 Fig: Direct comparison of total distribution volume (VT) in the thalamus for high affinity binders (HABs) and mixed affinity binders (MABs) calculated respectively with ABSS-IF, CS-IF and ICA-IF. S2.1 Fig: Bland-Altman plot of total distribution volume (VT) in cerebellum region. S2.2 Fig: Bland-Altman plot of total distribution volume (VT) in temporal cortex region. S2.3 Fig: Bland-Altman plot of total distribution volume (VT) in striatum region. S2.4 Fig: Bland-Altman plot of total distribution volume (VT) in thalamus region. S3 Fig: Log-Log scale plot of ABSS-IF and ICA-IF performed on carotid region. S4 Fig: Log-Log scale plot of the first and the second independent components. (DOC) [file pone.0115768.s002.doc]

**File S2: Supplementary figures**

Figure S1.1.

Figure S1.2.

Figure S1.3.

Figure S1.4.

Figure S2.1

Figure S2.2

Figure S2.3.

Figure S2.4


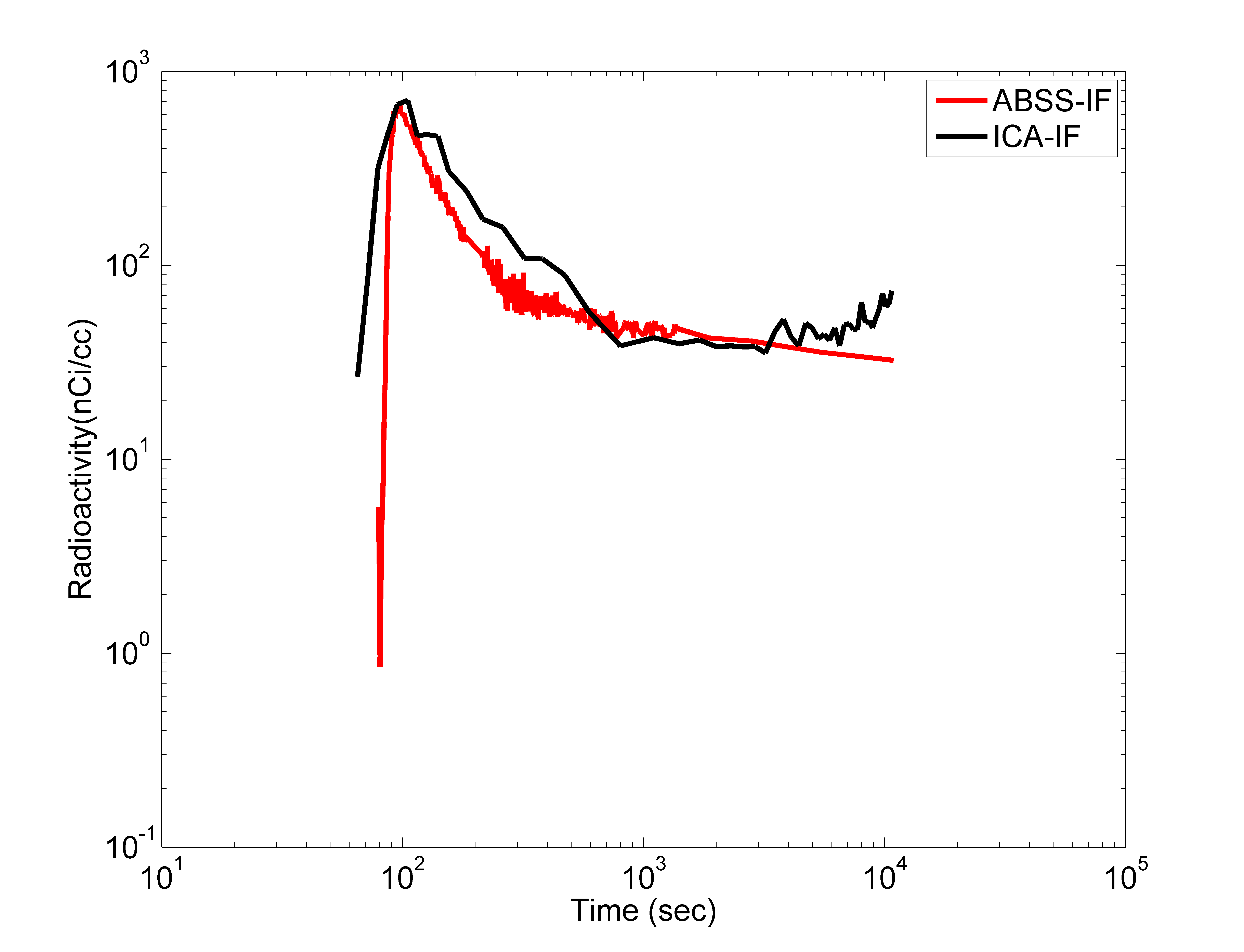


Fig S3


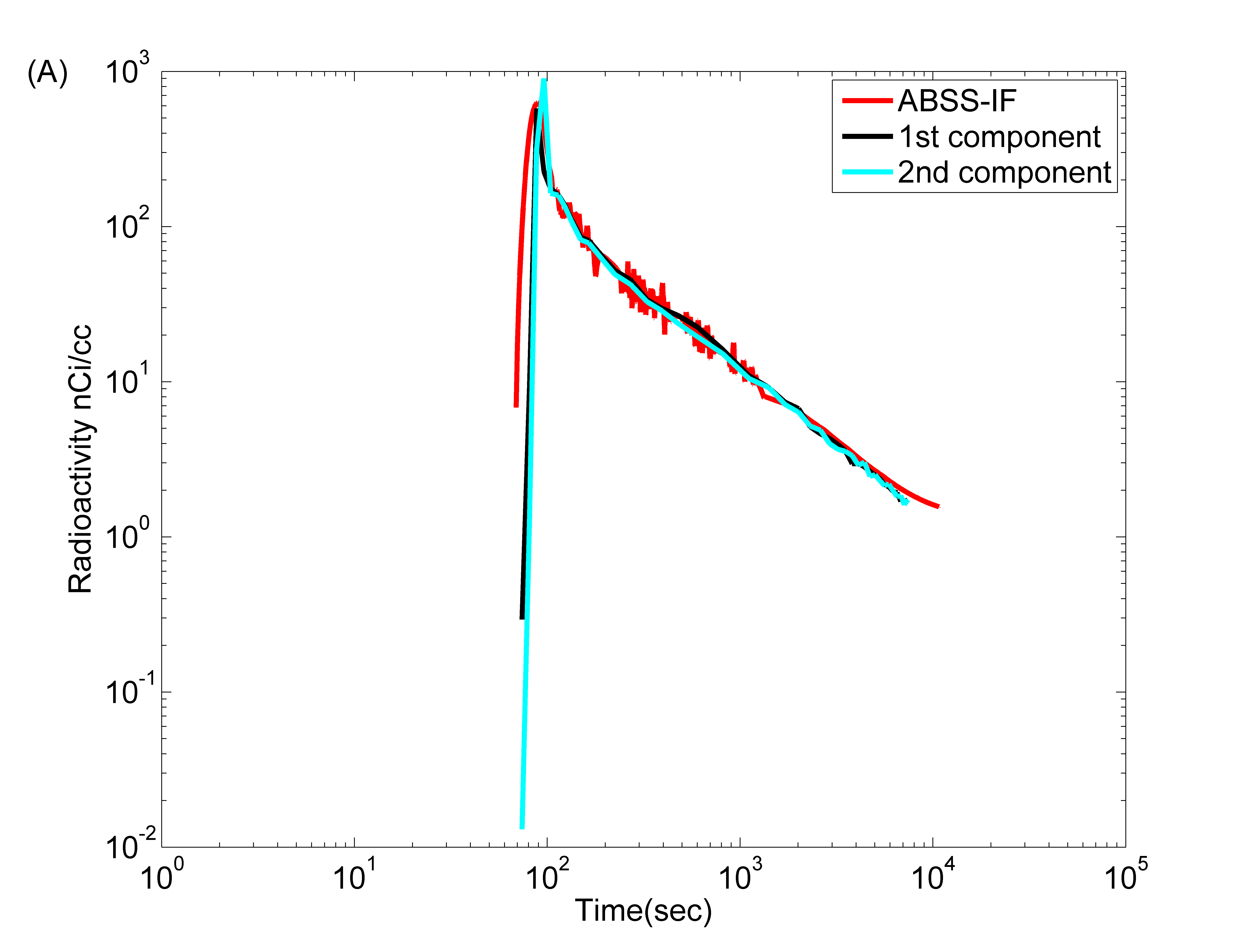


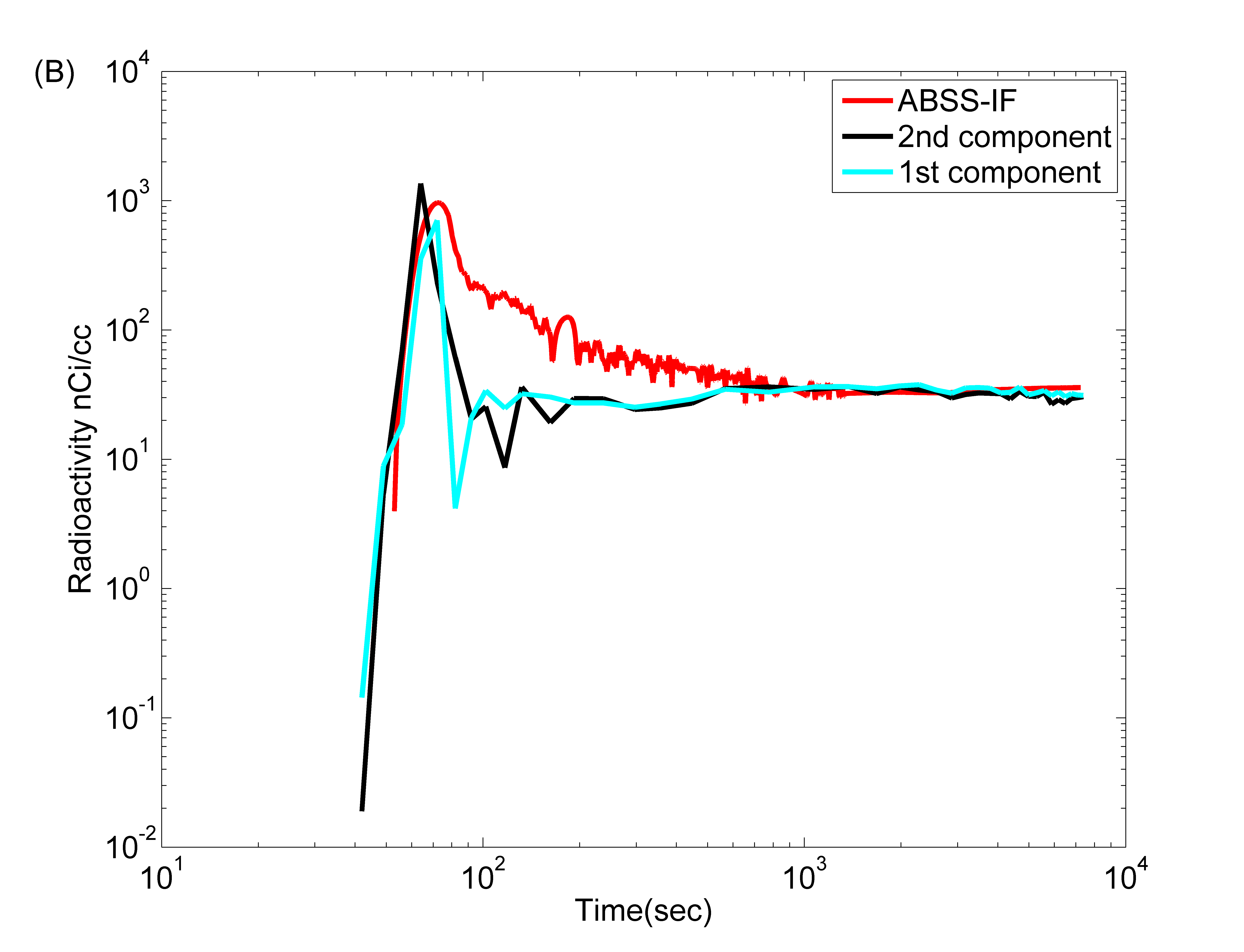


Figure S4.
